# 

**Published:** 1871-01

**Authors:** 


### CONTENTS.

ORIGINAL COMMUNICATIONS.

Thoughts on Respiration........ 9
Address........ 18
Ohio State Dental Society—Discussions........ 22

SELECTIONS.

Selections from a Clinical Lecture on Tumors........ 34
Physiology of the Pancreatic Secretion........ 36
A New Preparation of Cotton for Stanching Hemorrhage........ 37
Loss of Speech After Chloroform........ 38
Simple Method of Arresting Obstinate Epistaxis........ 38
Bee Stings........ 38
Antimonoid........ 38
Irregular Dentition........ 39
Tic Douloureux Cured by Galvanism........ 40
Neuralgia of the Jaw Bones........ 41
A Legion of Leeches........ 42
Skin Grafting........ 44
Immobility of the Jaws........ 45
An Itemized Bill........ 47
Neuralgic Pill........ 47
Oxygen Gas Prepared at the Ordinary Temperature........ 47
Corns on the Feet........ 48
Parchment Paper........ 48

EDITORIAL.

Bone Phosphate........ 49
The Forthcoming Volume........ 50
Table of Societies........ 51
Meetings........ 51
New Foil........ 52

